# Clinical application of electroacupuncture in enhanced recovery after surgery

**DOI:** 10.3389/fresc.2023.1135618

**Published:** 2023-05-24

**Authors:** Yu Mao, Lifang Yang

**Affiliations:** ^1^ Department of Cardiovascular Surgery, Xijing Hospital, Air Force Medical University, Xi’an, China; ^2^ Department of Anesthesiology, Xi'an Children Hospital, Xi'an, China

**Keywords:** electroacupuncture, enhanced recovery after surgery, perioperative period, clinical application, rehabilitation

## Abstract

Enhanced recovery after surgery (ERAS) is currently the recommended surgical strategy, the main content of which is to reduce perioperative stress response and postoperative complications through perioperative multimodal analgesia and intensive surgery. Since ERAS was introduced, many rehabilitation medicine teams have been deeply involved, including physical therapy, occupational therapy, nutrition therapy and psychological counseling. However, ERAS lacks several powerful means to address perioperative prognostic issues. Therefore, how to further improve the effects of ERAS, reduce perioperative complications and protect vital organ functions has become an urgent problem. With the continuous development of traditional Chinese medicine, electroacupuncture (EA) has been widely used in various clinical applications, and its efficacy and safety have been fully proved. Recent studies have shown that the application of EA in ERAS has had an important impact on rehabilitation researches. In terms of reducing complications, the therapeutic effects of EA treatment mainly include: reducing pain and the use of analgesics; Improvement of postoperative nausea and vomiting; Postoperative immune function treatment; Relieve anxiety and depression. In addition, EA also protects the recovery of physiological functions, including cardiovascular function, cerebrovascular function and gastrointestinal function, etc. To sum up, the complementary strengths of EA and ERAS will allow them to develop and combine. This review discusses the potential value and feasibility of EA in ERAS from the aspects of improving perioperative efficacy and protecting organ functions.

## 1. Introduction

Enhanced recovery after surgery (ERAS) refers to use a series of optimal perioperative interventions proven by evidence-based medicine to reduce psychological and physical traumatic stress responses, in order to facilitate rapid recovery of patients, reduce the risk of readmission and death and decrease costs. The concept of ERAS was first proposed by Kehlet in 2001, and was first applied in a gastrointestinal surgery (1). The core content is to reduce stress response and complications through multi-mode analgesia during perioperative period, to strengthen nutrition, get out of bed and carry out functional exercises as soon as possible after surgery, to reduce stress response and complications, so as to relieve the pain of patients and speed up recovery. In recent years, the application of ERAS has been gradually expanded to orthopedics, cardiothoracic surgery, obstetrics and gynecology, urology, general surgery and other fields. Clinical practice shows that the implementation of ERAS and related methods must be based on evidence-based medicine and multidisciplinary cooperation, which reflects the core concept of rapid recovery as the main goal, while also in consideration of the underlying diseases, types of surgery, perioperative complications and other specific conditions of patients.

Meanwhile, though ERAS can help patients achieve enhanced recovery, there is still great space for improvement in the treatment of post-operative ileus (POI), post-operative nausea and vomiting (PONV), and reduction of opioid dose. According to traditional Chinese medicine (TCM), acupoints are the specific parts of the meridians where qi and blood gather, and come in and out of the body surface for acupuncture, which are also the stimulation points of electroacupuncture (EA), and the reaction points of visceral physiological functions and pathological changes (2). EA is performed on the basis of traditional acupuncture, which is combined of acupuncture and electric stimulation technology, and the therapeutic effect has been confirmed by various of clinical and experimental studies (3). Studies have demonstrated that EA can improve neuromuscular activity, analgesia, immune regulation and regulate the functions of viscera and organs (3). EA is mainly to stimulate acupoints through the needle tail electrify, so as to achieve the purpose of treatment. Waveform, time, frequency and intensity are stimulus parameters of EA, among which frequency and intensity are the most important.

**Figure 1:**
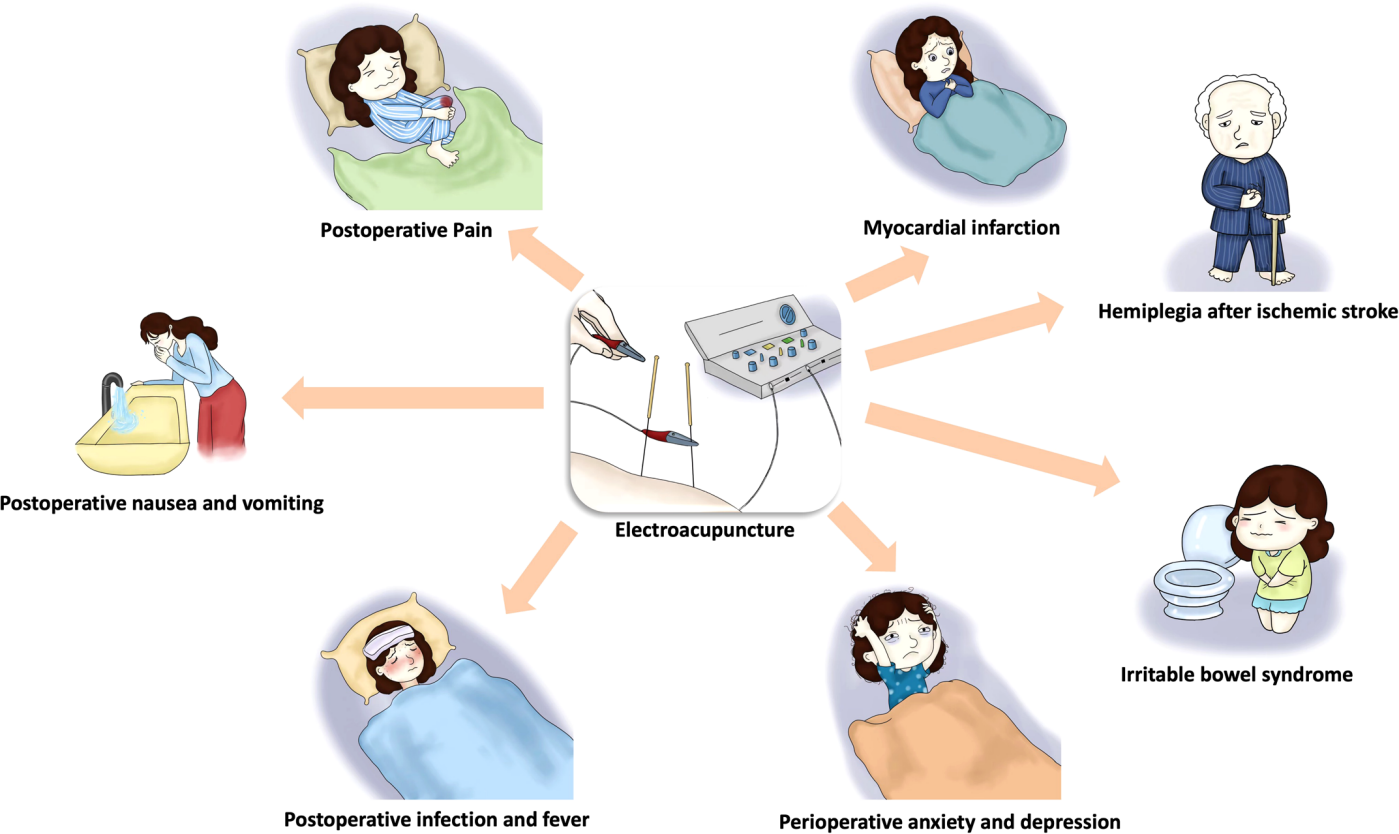
The main applications of electroacupuncture in enhanced recovery after surgery.

Overall, ERAS focuses on improving symptoms through physical therapy, reducing drug intake and accelerating physical recovery; While EA can reduce the amount of perioperative analgesics, decrease the occurrence of side effects, inhibit nausea and vomiting and accelerate postoperative recovery. Therefore, the combination of ERAS and EA can significantly reduce the incidence of adverse reaction and discomforts, stabilize hemodynamics (Figure 1). This review aims to explore the potential value and feasibility of EA in ERAS from promoting perioperative efficacy, improving postoperative immune status and protecting organ function.

## 2. The application of electroacupuncture in the perioperative period

**Figure 2:**
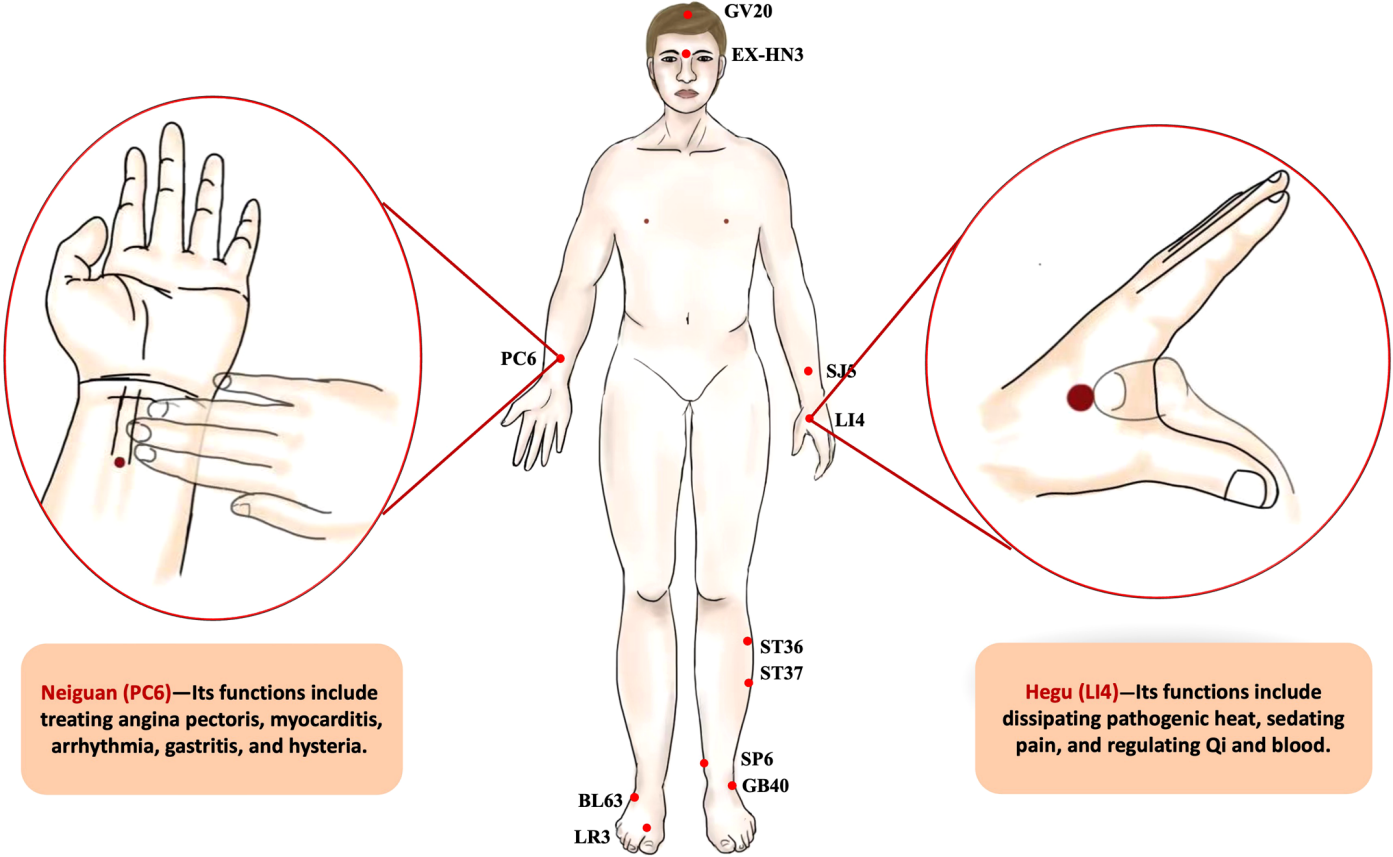
The acupoints act in the perioperative period. (**Central drawing**) (1) GV20 (Baihui): this point is located at the vertex where the three Foot-yang meridians, the liver meridian, and the Governor Vessel meet; (2) EX-HN3 (Yintang): this point lies at the midpoint of the line connecting the two eyebrows; (3) SJ5 (Waiguan): this point is on the three jiao meridian, located at 3.0 cun above the back of the wrist stripes; (4) ST36 (Zusanli): the point is on the legs, 3.0 cun below the knee; (5) ST37 (Shangjuxu): the point is on the legs, 3.0 cun below ST 36; (6) SP6 (Sanyinjiao): this point is at 3.0 cun above the medial malleolus, which is the intersection of 3 meridians (the spleen, liver, and kidney) and is located on the inner side of the calf; (7) GB40 (Qiuxu): this point is between the lateral malleolus and the peroneal trochlea of the calcaneus; (8) BL63 (Jinmen): This point is located in the foot lateral part, with the lateral malleolus front straight down, it is at the lower edge of the cuboid; (9) LR3 (Taichong): this point lies on the dorsum of the foot and the junction of the 1st and 2nd metatarsal bones. (**Left drawing**) Neiguan (PC6): this point is at 2.0 cun above the wrist stripes, between palmaris longus tendon and flexor carpi radialis tendon. Its functions include treating angina pectoris, myocarditis, arrhythmia, gastritis, and hysteria. (**Right drawing**) Hegu (LI4): this point is located on the dorsum of the hand between the first and second metacarpal bones in the first dorsal interosseous muscle on the radial aspect of the second metacarpal. The location of the point is depressed like a valley. Its functions include dissipating pathogenic heat, sedating pain, and regulating Qi and blood.

ERAS is essentially a conceptual innovation that challenges and corrects the traditional view of surgery based on evidence-based medicine. However, ERAS lacked an effective means to solve problems in the perioperative period, such as POI and PONV. Many researchers have paid attention to the important role of EA, especially in the perioperative period. Researchers have proposed a new concept of perioperative acupuncture medicine, which has aroused the attention in the clinic (4). In this section, we discuss the latest research progress of EA in the improvement of prognosis (Table 1). The main functioned acupoints are displayed in Figure 2.

**Table 1:** The prescribed dose of electroacupuncture treatment in reducing perioperative complications during enhanced recovery after surgery.

Aspects	Author (reference)	Disease/surgery type	Acupuncture points	Parameters of manipulation	Results/conclusion
EA applications in postoperative multi-mode analgesia	An et al. (5)	Patients after supratentorial tumor resection	LI4, SJ5, BL63, LR3, ST36, GB40	2/100 Hz, 3 s	EA improves the quality of postoperative analgesia, promotes appetite recovery and decreases dizziness and feeling of fullness in the head.
	Seevaunnamtum et al. (6)	Patients undergoing gynaecological surgery	PC6, LI4	2/15 Hz, 5 min	EA has an opioid-sparing effect and can reduce PONV.
	Hershman et al. (7)	Patients with early-stage breast cancer	-	30–45 min, once a week	EA results in a statistically significant reduction in joint pain
	Dalamagka et al. (8)	Patients with inguinal hernia	SP6, ST36, LI4, PC6, BL60, KI3	1/2 Hz, 40 + 60 min	EA reduces postoperative pain after surgery and decreases stress hormone levels and anxiety.
EA applications in prevention and treatment of postoperative nausea and vomiting	Alkaissi et al. (9)	Patients undergoing gynecological surgery	PC6	-	PC6 may have a place as prophylactic antiemetic therapy during gynecological surgery.
	Kim et al. (10)	Patients undergoing laparoscopic hysterectomy	PC6	1/50 Hz, 50 mA	PC6 can reduce PONV after laparoscopic hysterectomy
	Rusy et al. (11)	Patients undergoing tonsillectomy	PC6	4 Hz, 20 min	PC6 reduced the occurrence of nausea, but it did not significantly reduce the incidence or number of episodes of emesis or the use of rescue antiemetics.
	Gan et al. (12)	Patients undergoing major breast surgery	PC6	2/100 Hz, 30–60 min	EA is more effective in controlling nausea in comparison with ondansetron
	Ralston-Wilson et al. (13)	High-risk pediatric patients	-	4 days	Intraoperative multipoint EA may be a safe and efficacious adjunct for PONV in high-risk pediatric patients.
	Kim et al. (14)	Patients undergoing laparoscopic colorectal cancer resection	PC6, LI4, HT7, ST36, ST37, LR3, LI11, SP6, SP4, HT8, GB41	2–100 Hz, 20 min	PC6 can reduce PONV after laparoscopic colorectal cancer resection
EA applications in regulation of postoperative immune and anti-inflammatory functions	Meng et al. (15)	Patients with sepsis-induced intestinal dysfunction with syndrome of obstruction	ST36, ST37	4 Hz, 20 min	ST36, ST37 can reduce inflammatory reaction and has protective effects on intestinal function in patients with sepsis-induced intestinal dysfunction with syndrome of obstruction
EA applications in remission of perioperative anxiety and depression	Guo et al. (16)	Individuals with sub-syndromal depression	GV20, LR3, LI4, PC6, HT7, KI3, KI6, SP6, ST36	5–10 mA, 2/100 Hz, 30 min	Early EA intervention may alleviate depressive symptoms
	Wiles et al. (17)	Patients with preoperative anxiety	EX-HN3	30 min	EX-HN3 reduces preoperative anxiety levels in patients awaiting neurosurgery.

LI4, Hegu; SJ5, Waiguan; BL63, Jinmen; ST36, Zusanli; GB40, Qiuxu; PC6, Neiguan; SP6, Sanyinjiao; BL60, Kunlun; KI3, Taixi; HT7, Shenmen; ST37, Shangjuxu; LR3, Taichong; LI11, Quchi; SP4, Gongsun; HT8, Shaofu; GB41, Zulinqi; GV20, Baihui; KI6, Zhaohai; EX-HN3, Yintang.

### 2.1. Participation in postoperative multi-mode analgesia

Postoperative analgesia is one of the main aspects of ERAS, which includes adequate analgesia and minimizing opioid dose. Adequate postoperative analgesia can reduce excessive stress, help patients get out of bed as soon as possible, and promote patient recovery, which identifies as a potentially effective non-pharmaceutical treatment for pain. In addition, opioids are used to relieve postoperative pain through intravenous or local blockade, which are associated with many side effects, such as nausea and vomiting, intestinal obstruction, respiratory depression, constipation, and delayed postoperative recovery (18–21). At present, various studies have demonstrated that EA can significantly relieve pain and reduce opioid dose after cardiac surgery, craniotomy, total knee replacement (TKA), gynecological surgery, thyroid surgery, breast surgery and abdominal surgery. Le and colleagues conducted a randomized controlled trial (RCT) that investigated the effect of preoperative EA on opioid demand during routine cardiac surgery and concluded that EA may reduce fentanyl use during the surgery (22). Li et al. found that EA of Hegu (LI4) and Waiguan (SJ5), Jinmen (BL63) and Taichong (LR3), Zusanli (ST36) and Qiuxu (GB40) could improve postoperative analgesia quality and reduce the side effects of opioid in patients who underwent supratentorial tumor resection (5). In addition, EA has been used to improve recovery, and reduce some uncomfortable experiences, such as head satiety and dizziness (5). To investigate the benefits of EA in relieving pain in TKA patients, a study which enrolled 47 patients into different groups. The results showed that compared with the control group, the demand of patient-controlled analgesia (PCA) was significantly reduced in the EA group (23). Similarly, Chen et al. analyzed 17 RCTs and concluded that EA may be an effective auxiliary analgesia after TKA (24). In addition, researchers conducted a study on postoperative analgesia by EA in patients who underwent gynecological surgery, in which the EA group received 2 Hz stimulation in bilateral pericardial tract. By comparison with the control group, they concluded that the EA group were more likely to experience less pain (6). Lacobone and his colleagues found that patients in the EA group had lower remifentanil requirements and better performance in numerical rating Scales (NRS) and McGill scores, so they concluded that EA may be more helpful in relieving pain after thyroid surgery. In a multicenter, large-sample RCT, Hershman et al. studied the function of EA in 226 postmenopausal women with early breast cancer. The women, who took aromatase inhibitors and scored at least 3 on the Breif Pain Iventory for Worst Pain (BPI-WP), found that EA led to a statistically significant reduction in joint pain (7). In addition, EA was performed on patients with selective hernia surgery, which visual analogue scale (VAS) and other indicators were observed. The results showed that EA could reduce pain after inguinal hernia repair (8). Above all, the success of EA depends on several factors, such as the acupoint selection, the opportunity of EA, duration of treatment. Therefore, based on the clinical perspective of ERAS, EA may lead to better control of pain and less use of analgesics, including opioids, to accelerate recovery.

### 2.2. Prevention and treatment of postoperative nausea and vomiting

Postoperative nausea and vomiting (PONV) is a common complication after surgeries, which may lead to dehydration, electrolyte imbalance and delayed discharge (25). Despite the widespread application of prophylactic antiemetic agents, minimally invasive surgical techniques and short-term anesthetics, PONV still affects 20%–40% of surgical patients, and the incidence of some high-risk patients is up to 80% (26). Postoperative analgesia with opioids may cause PONV, while the dual effects of antiemetic and analgesic of EA can reduce adverse reactions (27). Studies have shown that multiple stimulations of Neiguan (PC6) can effectively prevent PONV (9, 10). In addition, the most commonly used acupoint in most PONV studies is PC6. The effect of EA on the prevention of PONV is similar to that of commonly used drug therapy (11). The effect of EA is better than intravenous infusion of ondansetron, and the combined use may also enhance the antiemesis effect of ondansetron (12). An et al. used EA of LI4, SJ5, BL63, LR3, ST36 and GB40 to stimulate patients with craniotomy, and the results showed that the above acupoints could significantly reduce the incidence of PONV (5). Ralston-Wilson et al. concluded that EA may be a safe and effective auxiliary method for postoperative treatment of PONV in high-risk pediatric patients (13). Similarily outcomes were found in Kim's research (14). Lee and his colleagues showed that stimulation of PC6 had similar effects to the use of antiemetic drugs, which could reduce the incidence of PONV and the utilization of postoperative antiemetic measures (28). The clinical efficacy of EA has been recognized by researchers at home and abroad, but the mechanism of EA in the treatment of PONV is still not clear, which restricts its clinical promotion. Some researchers believe that PONV may be related to the imbalance of gastrointestinal hormone secretion, and EA has a good effect on regulating motilin and serum gastrin levels, thus reducing the incidence of PONV (29). A research showed that EA plays a role in the treatment of PONV through its influence on the vagus nerve (30). Some researchers showed that EA may treat PONV by activating adrenergic and norepinephrine to change the transmission of 5-hydroxytryptamine (5-HT) (31). Future clinical researches with large samples are needed to carry out, and further research on the mechanism of EA in the treatment of PONV is necessary as well.

### 2.3. Regulation of postoperative immune and anti-inflammatory functions

Perioperative stress response refers to the complex interaction between nerve, endocrine, immune and coagulation system when patients are strongly stimulated during perioperative anesthesia. It is the non-specific defense response to external stimulation. If the stress response is not reduced, the surgery is often used as an important stressor and may cause various of harm. Therefore, it is important to use anesthetic and non-anesthetic measures to reduce harmful stress responses during the perioperative period. In addition, inflammation is a defensive response to stimulations. Hence, how to reduce and control perioperative stress and trauma, to promote organ function to normal as soon as possible, to shorten postoperative hospital stay and to improve prognosis is the core content. In recent years, a large number of studies have found that EA can play an anti-inflammatory role. Meng et al. performed EA of ST36 and Shangjuxu (ST37) to sepsis patients, respectively. Based on routine intensive care units (ICU) treatment, pretreatment twice daily for 5 days was observed to significantly reduce TNF-α and IL-10 levels in the EA group at 1, 3 and 7 days after treatment compared to the control group. They believed that EA could attenuate the inflammatory response and the stress response (32). This may be related to the release of immunomodulatory factors stimulated by EA, such as corticosteroids and β-endorphins. Similar findings were found in Meng's study (15). In addition, Zhang et al. found that EA may exert anti-inflammatory effects by influencing the hypothalamic-pituitary adrenal (HPA) axis to reduce the levels of cyclooxygenase-2 (COX-2) and prostaglandin E2 (PGE2), and enhancing the sympathetic nervous system to induce peripheral opioid release (33). Similarily, EA stimulates the adrenal gland to release catecholamine and acts on peripheral dopamine D1 receptors to produce systemic anti-inflammatory effects (34). Meanwhile, researchers also indicate that EA can exert anti-inflammatory effects via the cholinergic anti-inflammatory pathway (35, 36). However, the effects of EA on sympathetic and parasympathetic nerves have not been fully understand and probable inconsistent in different acupoints or different diseases, further researches need to be carried out at a deeper level.

### 2.4. Remission of perioperative anxiety and depression

Patients after surgeries are prone to anxiety and depression. Once anxiety or depression occurs, the psychological burden of patients is often increased and the postoperative recovery will be delayed, which is not in line with ERAS (37, 38). Severe anxiety and depression can affect the patient's ability to carry out normal activities, and patients with generalized anxiety disorder (GAD) have prominent anxiety that is persistent and inappropriate. In addition, symptoms, including physical and mental symptoms, may last for months, recurs periodically and are exacerbated by stressful events (39). Previous studies have found that EA can ease anxiety and depression in such patients, and accelerate the recovery process. In a single-center RCT, patients in the EA group received stimulation of Baihui (GV20), Sanyinjiao (SP6) and LR3, and it was concluded that EA was the ideal choice for depression treatment (38). Guo et al. found in a study of patients with subsyndromic depression (SSD) that EA can help relieve anxiety and depression (16). Zhang et al. summarized the studies on EA to treat depression, and proposed that the early use of selective serotonin reuptake inhibitors (SSRIs) and EA for primary depression is more effective than SSRIs alone, and can control depressive symptoms better and earlier (40). Similarily, Amorim and his colleagues showed that whether clinicians chose the intensity, frequency, and acupoint of EA, all can reduce patients' anxiety levels (41). In a prospective RCT, patients simulatyed of Yintang (EX-HN3) before neurosurgery, the results showed that EX-HN3 may reduce the preoperative anxiety level of patients (17).

## 3. Protection of vital organ function

**Figure 3:**
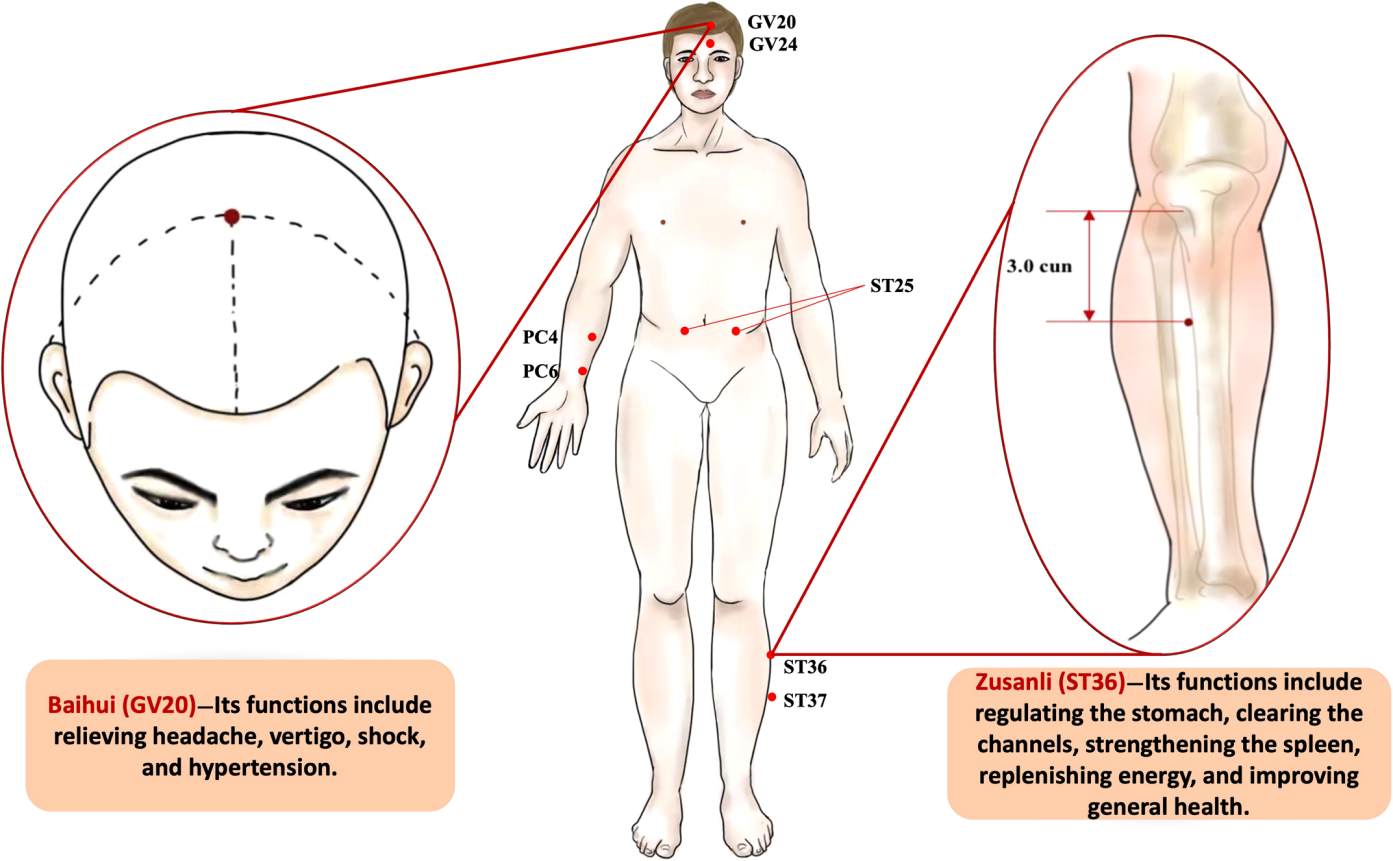
The acupoints applied in the protection of vital organ functions. (**Central drawing**) (1) GV24 (Shenting): this point is at 0.5 cun before the hairline, which is also in the upper position of the forehead. (2) PC4 (Ximen): this point is at 5.0 cun above the wrist stripes, on the palmar side of the forearm. (3) PC6 (Neiguan): this point is at 2.0 cun above the wrist stripes, between palmaris longus tendon and flexor carpi radialis tendon. (4) ST 25 (Tianshu): this point is over the middle of the stomach. (5) ST37 (Shangjuxu): the point is on the legs, 3.0 cun below ST 36. (**Left drawing**) Baihui (GV20): this point is located at the vertex where the three Foot-yang meridians, the liver meridian, and the Governor Vessel Dumai meet. This point is closely connected with the brain, and it is the key point to regulate the brain function, for regulating the body of Yin and Yang balance plays an important role. Its functions include relieving headache, vertigo, shock, and hypertension. (**Right drawing**) Zusanli (ST36): this point is located on the anterior aspect of the leg in the tibialis anterior muscle, 3.0 cun inferior to the lateral depression underneath the knee cup and one fingerbreadth lateral to the tibial crest. Its functions include regulating the stomach, clearing the channels, strengthening the spleen, replenishing energy, and improving general health.

EA is often regarded as a hallmark of TCM, which may achieve analgesic effects by regulating the release of peptido-based neurotransmitters. Numerous studies have confirmed that EA has unique advantages for surgical patients which often beyond the scope of traditional anesthesia. Preoperative EA can improve preoperative status and reduce the use of anesthetics, while surgical EA can protect cardiovascular and cerebrovascular function, help postoperative intestinal function recovery and shorten hospital stay, which makes ERAS further prominent (Table 2). Here, the mainly functioned acupoints in protecting vital organs are shown in Figure 3.

**Table 2:** The prescribed dose of electroacupuncture treatment in the protection of vital organs during enhanced recovery after surgery.

Aspects	Author (reference)	Disease/surgery type	Acupuncture points	Parameters of manipulation	Results/conclusion
EA applications in protection of cardiovascular function	Wang et al. (42)	Patients undergoing percutaneous coronary intervention	PC6, PC4	30 min	EA prior to percutaneous coronary intervention significantly reduced cTnI release and protected patients from subsequent myocardial injury after procedure.
	Yang et al. (43)	Patients with acquired heart valve diseas	PC6, LU7, LU2	0.8–1.9 mA, 5/30 Hz, 30 min	EA may alleviate cardiac ischemia-reperfusion injury in adult patients undergoing heart valve replacements.
	Yu et al. (44)	Patients undergoing heart valve replacement with cardiopulmonary bypass	PC6, PC4, GV24, GV20	0.5–1.2 mA, 2/100 Hz	EA reduced the occurrence of complications and played a role of cardioprotective effect
	Ni et al. (45)	Patients with congenital heart defects	PC6	2 Hz, 30 min	PC6 acupoint is effective for attenuation myocardial injury (reduction in cTnI and C-reactive protein level).
	Liu et al. (46)	Patients with prehypertension and stage I hypertension	ST36, PC6, LR3, SP4, LI11	20 min	Acupuncture might lower blood pressure in prehypertension and stage I hypertension.
EA applications in protection of brain function	Qi et al. (47)	Patients with foot drop after stroke	GB34, ST36, GB37, GB39, SP6	30 min, once a day	Acupuncture combined with rehabilitation achieves better effect than simple rehabilitation for foot drop after stroke.
	Yang et al. (48)	Patients with vascular cognitive impairment no dementia	ST36, SP10, RN17, RN12, RN6, GV20, GV16, BL15, BL45, HT5, KI6, KI3, GB39, ST40, PC6, BL17	30 min	Compared with citicoline, acupuncture has comparable and even superior efficacy with improved cognitive and daily living performance as a complementary and alternative medicine treatment.
	Zhang et al. (49)	Patients scheduled spine surgery	GV20, GV14, ST36	1 mA, 2/15 Hz	EA reduced the serum concentration of IL-6, IL-10, and S100β at 1 h after skin incision.
	Lin et al. (50)	Patients with vascular cognitive impairment no dementia	GV20, GV24	30 min	EA treatment as an ideal choice for vascular cognitive impairment no dementia.
EA applications in protection of gastrointestinal function	Zhang et al. (51)	Patients undergoing elective open resection of malignant colorectal tumors	ST36	2 Hz, 30 min	EA at ST36 accelerates the recovery of gastrointestinal motility after colorectal surgery.
	Liu et al. (52)	Patients undergoing vascular surgery	PC6, ST36, ST37	5–10 mA, 2/100 Hz, 30 min	EA is an effective method for the prevention of postoperative gastrointestinal dysfunction
	Zheng et al. (53)	Patients with refractory functional dyspepsia	ST36, PC6, LR3, ST2, SP4, SP9	0.1–1 mA, 2–100 Hz, 30 min	Acupuncture efficaciously improves dyspeptic symptoms in patients with refractory functional dyspepsia.
	Xu et al. (54)	Patients with functional dyspepsia	ST36, PC6	4 mA, 25 Hz	EA at the ST36 and PC6 points accelerates solid gastric emptying in FD patients with delayed gastric emptying and relieves dyspeptic symptoms in FD patients with normal gastric emptying.
	Wang et al. (55)	Patients with postprandial distress syndrome	PC6, ST25, ST36, SP4, GV20, RN17, RN12, RN6, SP3	30 min	EA tended to improve symptoms and the quality of life among patients with postprandial distress syndrome.
	Shi et al. (56)	Patients with irritable bowel syndrome	ST25, ST37	3 mA, 2 Hz, 30 min	EA was effective in improving IBS symptoms and modulate abnormal expressions of 5-HT, 5-HT3R, and 5-HT4R in the colonic tissue.

ST36, Zusanli; PC6, Neiguan; SP6, Sanyinjiao; KI3, Taixi; ST37, Shangjuxu; LR3, Taichong; LI11, Quchi; SP4, Gongsun; GV20, Baihui; KI6, Zhaohai; PC4, Ximen; LU7, Lieque; LU2, Yunmen; GV24, Shenting; GB34, Yanglingquan; GB37, Guangming; GB39, Xuanzhong; SP10, Xuehai; RN17, Danzhong; RN12, Zhongwan; RN6, Qihai; GV16, Fengfu; BL15, XInshu; BL45, Weicang; HT5, Tongli; ST40, Fenglong; BL17, Geshu; GV14, Dazhui; ST2, Sibai; SP3, Taibai; SP9, Yinlingquan.

### 3.1. Protection of cardiovascular function

Cardiovascular diseases have become the major diseases threatening human health. In addition to medication, many serious cardiovascular diseases require interventions. Therefore, the protection of cardiovascular function after interventions has become a hot topic, and many researchers have carried out studies on the protective effect of EA.

#### 3.1.1. Protection of myocardial function

In recent years, the perioperative use of EA has brought great benefits to the patients who have cardiovascular diseases. In a prospective RCT, 388 patients underwent percutaneous coronary intervention (PCI) were divided into the control group and the EA group. Patients in the EA group received the stimulation of PC6 and Ximen (PC4) for 30 min prior to PCI. The results showed that the incidence of MI4a [cardiac troponin (cTn) I ≥ 0.20 ng/ml] at 24 h after PCI in EA group was lower than that in the control group, and the cardiac function was significantly improved. Therefore, they believe that EA before PCI may reduce the release of cTn I and protect patients from myocardial injury after PCI (42). In Yang's study, 60 patients with the valvular heart disease were divided into the EA group and the control group, and the length of ICU stay in the EA group was shorter than that in the control group. Meanwhile, at 6, 12 and 24 h after reperfusion, cTn I level in the EA group was significantly lower than that in the control group, suggesting that EA played a potential myocardial protective role (43). In another study, 44 patients underwent heart valve replacement were randomly assigned to the EA group and the control group, with patients of the EA group receiving the stimulation of PC6, PC4, Shenting (GV24), and GV20, respectively. They focused on the arrhythmia, recorded the time of first exhaustion and ambulation and counted the incidence of PONV. The results showed that EA not only played an important role in cardiac protection, such as anti-arrhythmic protection, but also reduced the incidence of PONV, shortened the time of first exhaustion and ambulation and thus decreased postoperative hospital stay (44). In another heart study, children aged 2–12 years old with congenital heart disease (CHD) who needed surgery were divided into the treatment group and the control group. In the treatment group, EA was simulated of PC6 for 30 min after basic anesthesia. After a long period of follows-up, cTn I level in the treatment group decreased significantly, and the ventilation time and ICU time in the control group were significantly longer than those in the treatment group (45). In conclusion, EA is effective in alleviating postoperative myocardial injury.

#### 3.1.2. Maintain blood pressure stable

Primary hypertension is a major risk factor for cardiovascular diseases and ischemic stroke (57). Many elderly patients before undergoing elective surgery often have hypertensions, which brings some difficulty to perioperative anesthesia management. Fluctuations of blood pressure may have adverse consequences for patients, such as unstable blood circulation, malignant hypertension, shock and heart failure. In view of the many side effects of drugs, in addition to reduce the usual drug dosage, EA can also assist to achieve the effect of droping blood pressure, so as to achieve the purpose of reducing drug dose. Chen et al. summarized the findings of 30 related studies and found that EA can enhance the therapeutic effect of antihypertensive drugs (58). In another study, patients with mild hypertension received EA, and the results showed that EA could reduce the blood pressure, which may be related to the influence of sympathetic nerve by EA (46). Therefore, for patients with mild hypertension, EA play an auxiliary role in reducing the blood pressure.

### 3.2. Protection of brain function

Cerebral circulation carries blood to the brain through a dedicated network of the blood vessels. A healthy brain can regulate cerebral blood flow (CBF) in response to any physiological or pathological challenge. The brain is protected by its self-regulatory mechanisms. A growing number of studies suggest that “non-drug” approaches may provide new opportunities for the treatment of central diseases such as ischemic stroke, in which EA plays an important role in protecting brain function (59).

#### 3.2.1. Improvement of hemiplegia after ischemic stroke

Ischemic stroke is considered to be the leading cause of death and disability worldwide (60). Post stroke hemiplegia is one of the main functional disorders in ischemic stroke patients. Paralysis and spasm are common in ischemic stroke, most limbs pwith flaccid hemiplegia in the early stages after stroke. The upper neurons lose control of the lower neurons, and the uninjured center of the spinal cord goes into shock, resulting in flaccid hemiplegia, namely Brunstrom I–II. Natural recovery in flaccid hemiplegia lasts about 2 weeks. Reconstruction of motion is the key to ischemic stroke patients. However, the longer the duration of hemiplegia, the worse of the prognosis and the higher rate of residual physical disability (61). Therefore, the main objective of treatment is to reduce the duration of flaccid hemiplegia. At present, the main treatments after ischemic stroke include exercise rehabilitation, non-invasive brain stimulation, transcranial magnetic stimulation and physical therapies. However, all of the above treatments have various disadvantages, and EA is internationally recognized as the basic treatment for ischemic stroke-related diseases (62, 63). World Health Organization (WHO) recommended EA as an alternative and complementary strategy for ischemic stroke treatment (64). The advantages of EA in treating ischemic stroke have recently been highlighted in studies (65–67). Results of several RCTs showed that patients with flaccid hemiplegia after ischemic stroke in the EA group had better recovery in various evaluation criteria. In addition, when ischemic stroke patients are in hemiplegia, EA can promote the recovery of patients' muscle strength and muscle tension, improve the motion function of limbs, and effectively prevent the occurrence of various complications (47, 68–72). These findings suggest that EA, as a complementary therapeutic method, is effective and short-term safe for patients with post-stroke flaccid hemiplegia. However, methodological deficiencies in previous studies have led to the need to carefully design larger studies to confirm the potential benefits of EA for rehabilitation in patients with flaccid hemiplegia after ischemic stroke. In addition, EA also can prevent the risk of postoperative stroke. Wu et al. proved that EA can enhance cerebral glucose metabolism assessed by 18F - fluorodeoxyglucose/positron emission tomography (18F-FDG/PET) imaging to prevent propagation of tissue damage and improve neurological outcome in rats subjected to ischemia and reperfusion injury (73). However, further studies need to be conduct to explore the mechanisms and the real-world effect of EA treatment in prevention of stroke.

#### 3.2.2. Improvement of cognitive dysfunction

Cognitive dysfunction mainly refers to the impairment of patients' memory, executive power, spatial structure, computing power, attention and orientation, which ultimately leads to the loss of patients' ability to live (74). In the treatment of cognitive dysfunction, drug therapy is generally advocated, such as calcium antagonists, cholinesterase inhibitors and excitatory amino acid antagonists, which may delay the progression of patients' disease to a certain extent. However, the efficacy is limited, and the adverse reactions gradually increase with the extension of medication time (75). TCM has has rich clinical experience, especially EA to cognitive dysfunction is remarkable. EA can improve hemodynamics, reduce inflammatory response and enhance cognitive function of patients by stimulating acupoints in different parts of the body (48, 76). In Zhang's study, 90 patients before spinal surgery were randomly divided into the control group and the EA group, the EA group received EA of GV20 and ST36, respectively. The results showed that the levels of IL-6, IL-10 and S100β were lower in the EA group, so EA may improve postoperative cognitive function and has an effective protective effect in the brain (49). In a similar study, EA simulated of GV20 and ST36 was used to detect IL-6, IL-10, and S100β, and Mini-mental State Examination (MMSE) scores were recorded to evaluate cognitive function in 90 elderly patients underwent spinal surgery. The results showed that MMSE score in the EA group was higher than that in the control group, and IL-6, IL-10 and S100β levels were lower than that in the control group, suggesting that EA may improve postoperative cognitive function and reduce inflammatory response (49). Lin et al. divided 140 patients with vascular cognitive disorder into the control group and the EA group, and used Montreal Cognitive Assessment (MoCA) to evaluate the cognitive function of the two groups of patients (50). The results showed that MoCA score of the EA group was higher than that of the control group after treatment, which suggested that EA may improve the cognitive level of patients with vascular cognitive disorder. Wang et al. analyzed the clinical data of 50 patients with transient cerebral ischemia, and found that patients with vascular cognitive dysfunction of cerebral blood flow was significantly lower than the patients with non-vascular cognitive dysfunction, and the average blood flow negatively correlated with MoCA score (77). Studies above demonstrated that EA had an important role in improving cognitive dysfunction of relevant patients.

### 3.3. Protection of gastrointestinal function

Gastrointestinal function is one of the most important physiological functions in human body, which may affect postoperative recovery, increase postoperative complications and prolong postoperative hospital stay. However, perioperative EA has a good effect on regulating gastrointestinal function. According to a basic study, ST36 may accelerate colonic transport and stimulate distal colonic mosis through parasympathetic and cholinergic pathways (78, 79). Researchers suggested that stimulations of ST36 could help restore gastric function (51). In another clinical study, with the stimulation of PC6, ST36 and ST37 in patients underwent vascular surgery, the results showed that EA in ST36, PC6 and ST37 was beneficial to prevent postoperative gastrointestinal dysfunction. Meanwhile, EA can reduce the length of hospital stay for abdominal distension (52). Coincidentally, Zhang et al. performed stimulation of ST36 on patients who had elective colorectal surgery, which significantly shortened the postoperative exhaust time, thus accelerating the recovery of gastrointestinal dynamics after open resection of colorectal cancer (51). The improvement of gastrointestinal function by EA stimulation was consistent with ERAS. In addition, EA has a significant therapeutic effect on functional dyspepsia (FD) and irritable bowel syndrome (IBS).

#### 3.3.1. Improvement of functional dyspepsia

According to Roma IV classification, FD is the presence of upper abdominal symptoms, upper abdominal pain, upper abdominal burning, postprandial fullness and no evidence of structural disease on routine clinical examinations. EA treated for FD has been extensively studied. A recent RCT involving 3 hospitals in China evaluated the efficacy of EA in 200 patients with FD. The results showed that the effect was more significant in the EA group (53). Similarly, Ko and his colleagues conducted a study, which showed that gastrointestinal function was significantly improved after stimulation of PC6 and ST36 (54). In addition, a study comparing the efficacy of EA with different frequency in patients with postprandial depressive syndrome. The comparison results showed that the EA group showed better results at all time cut-off points (55). A large network meta-analysis (NMA) in 2017 included 22 RCTs that assessed the efficacy of EA and related therapies versus pro-dynamic therapy in patients with FD according to Roma IV classification. The results showed that EA combined with cephalosporin was significantly more effective in the treatment of FD than EA alone or cephalosporin alone. Therefore, Ho et al. concluded that the addition of EA to cephalosporin is likely to be the most effective treatment for FD (80).

#### 3.3.2. Treatment of irritable bowel syndrome

IBS is a common clinical condition characterized by recurrent abdominal pain that accords with Roma IV classification (81). Among patients involved by functional gastrointestinal diseases, patients with IBS may be the most likely to recurrent symptoms. However, several studies have investigated the potential benefits of EA with regard to IBS. First of all, EA can effectively regulate the function of vagus nerve, thus regulating the gastrointestinal function. Murakami et al. observed the effect of vagus nerve stimulation on gastrointestinal peristalsis in animal models and found that vagus nerve stimulation could improve gastric emptying and reduce postoperative pain (82). Another study on the effects of EA at abdominal and lower extremity acupoints on gastrointestinal movement, which found that EA promoted jejunum movement by stimulating parasympathetic pathways (83). Secondly, EA has regulatory effects on intestinal intermuscular nerve plexus, intestinal ganglion cells, Cajal interstitial cells, neurotransmitters and their receptors. Liang et al. found that EA treatment can alter excitatory and inhibitory neurons and restore the coordination between intestinal contraction and relaxation (84). Thirdly, EA was demonstrated to improve gastrointestinal motility, which may be related to decreased levels of gastrointestinal vasoactive intestinal peptide and calcitonin gene-related peptide (85). These studies have confirmed that EA may regulate intestinal movement through a variety of mechanisms. Currently, a meta-analysis conducted by the Cochrane group showed that EA was found to be significantly more effective than medication (antispasmodic drugs) in improving symptom severity. MacPherson and his colleagues conducted a study of a large group of patients affected by IBS in the United Kingdom. They demonstrated that patients who received conventional therapy in combination with EA reported statistically significant reductions in IBS symptom severity scores at 3, 6, 9, and 12 months compared with the control group (86). In another study, 85 patients with IBS were compared with stimulation of ST36 and ST37 once daily for 4 days. 37 gastrointestinal symptoms and stool consistency were assessed using Visual Analogue Pain Scale (VAPS) and Bristol Stool Scale (BSS). In addition, colonoscopy and biopsies were performed before and after treatment to assess the expression of vasoactive intestinal peptide (VIP), E neuropeptide and substance P (SP), which appears to function in the brain-gut axis, causing abnormal mucosal increase in patients with IBS. As in previous studies, both treatments improved gastrointestinal functions, especially in patients with IBS-C, where EA was more effective than moxibustion. In addition, the expression of colonic mucosal associated SP and VIP was significantly decreased in both groups. A study in 2015 investigated the efficacy of Tianshu (ST25) and ST37 in the treatment of patients with IBS through Visual Analogue Scale for Irritable Bowel Syndrome (VAS-IBS) (56). The VAS-IBS scores of both groups were significantly improved after 4 weeks of EA. In particular, EA showed better results in patients with IBS-C.

## 4. Limitations and future directions

**Figure 4:**
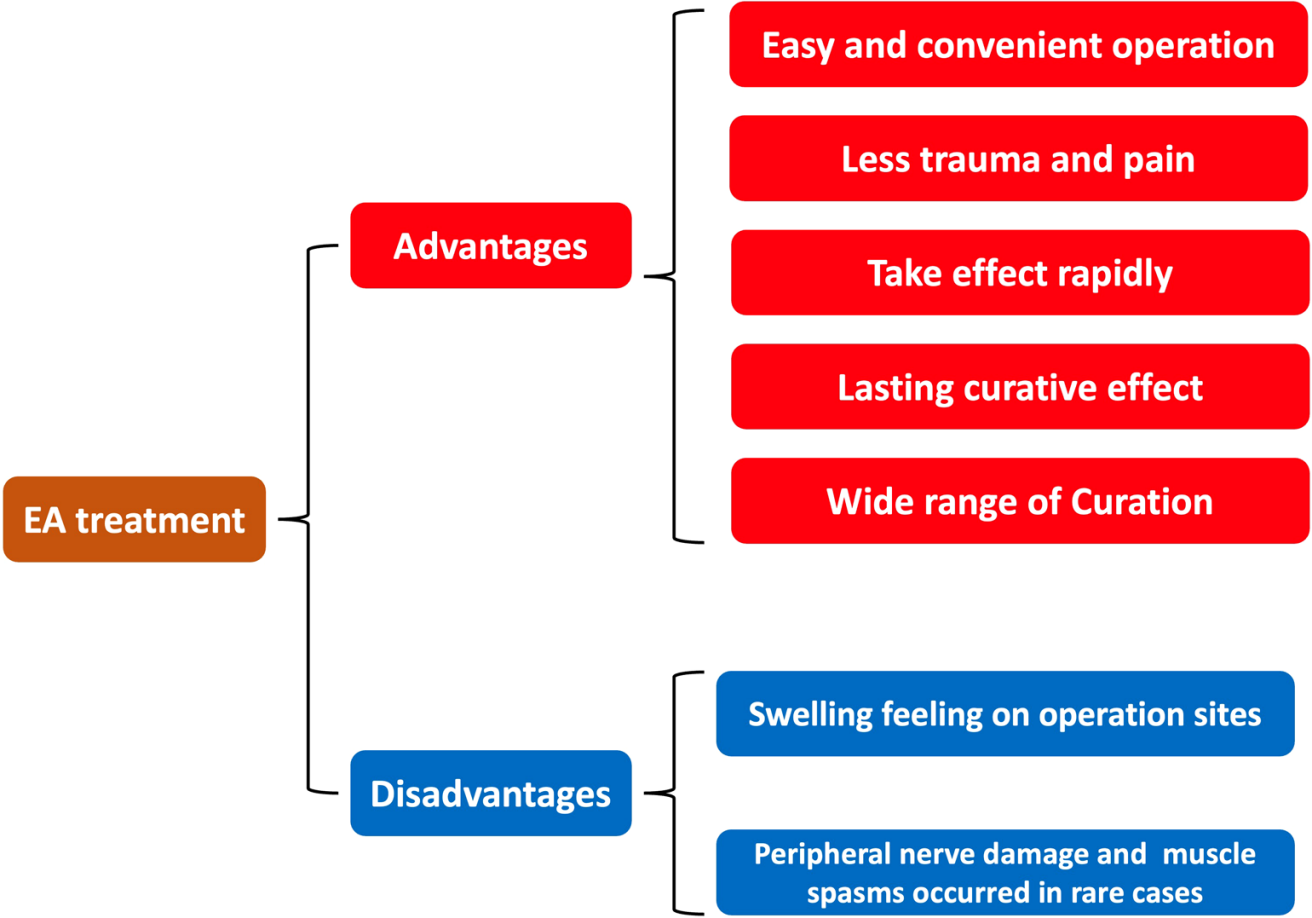
The advantages and disadvantages of electroacupuncture in enhanced recovery after surgery.

EA has been widely used in clinical practice, so that the quality of rehabilitation has been greatly improved, its simple, painless, convenient and effective means have been received more and more clinicians' recognition, researchers are committed to studying the mechanism of EA promoting postoperative rehabilitation. However, the theoretical system of EA in clinical application is not systematic enough, so that the application scope is limited. In addition, the application research in EA has been carried out for many years, the clinical value has been proved without popularized. Investigate the reason, one is the mechanism of EA is not fully clear (87); Secondly, it is difficult to change the traditional ideas which have occurred in the promotion of ERAS (88); Thirdly, the basic research of EA is seriously disjointed from clinical application, and its results are difficult to promote and improve clinical efficacy (87). Fourthly, there are some contraindications on EA treatment, mainly including: for weak resistance and elderly patients, it is better not to use EA (3, 24); Furthermore, if the patient is in a state of poor nutrition after operations, EA may cause intolerance or other postoperative complications (11, 13); In addition, for areas near the medulla or spinal cord of patients, the EA current should be turned down (65, 66). Last but not least, EA therapy has the following side effects: Generally, after EA treatment, there may be swelling feeling on operation sites (89). This feeling will linger for a while after the treatment is over and the needle is removed. This phenomenon is often seen in patients who are nervous or on their first treatment. In addition, EA may, in rare cases, cause side effects such as peripheral nerve damage, muscle spasms, and heart attacks, etc. (90). With the continuous development of modern medicine, EA will be analyzed and improved in the multi-disciplinary communication. With the guidance of TCM theory and combined with the scientific research achievements of modern medicine, both research results and clinical practice of EA will achieve significant improvement, and EA may play an increasingly important role in rehabilitation. Overall, despite with physical therapy, occupational therapy, nutrition and psychological counseling, etc. have achieved relatively satisfied results in prognosis, EA treatment may reply on its unique advantages to play an irreplaceable role in ERAS period (Figure 4).

## 5. Conclusion

ERAS has shown significant advantages over traditional perioperative management, reducing hospital stay, hospital costs, stress response and postoperative pain. The application of EA has shown remarkable effects in ERAS, so it is more and more widely used in perioperative period, bringing great benefits to patients. It is believed that EA will play an increasingly important role in the rehabilitation and the treatment in the future.
